# Utility and limitations of PHQ-9 in a clinic specializing in psychiatric care

**DOI:** 10.1186/1471-244X-12-73

**Published:** 2012-07-03

**Authors:** Takeshi Inoue, Teruaki Tanaka, Shin Nakagawa, Yasuya Nakato, Rie Kameyama, Shuken Boku, Hiroyuki Toda, Tsugiko Kurita, Tsukasa Koyama

**Affiliations:** 1 Department of Psychiatry, Hokkaido University Graduate School of Medicine, North 15, West 7, Kita-ku, Sapporo, 060-8638, Japan

## Abstract

**Background:**

The Patient Health Questionnaire-9 (PHQ-9), despite its excellent reliability and validity in primary care, has not been examined for administration to psychiatric patients. This study assesses the accuracy of PHQ-9 in screening for major depressive episode and in diagnosing major depressive episode in patients of a psychiatric specialty clinic.

**Methods:**

We compared operational characteristics of PHQ-9 as a screening and diagnostic instrument to *DSM-IV-TR* diagnosis by a trained psychiatrist as a reference standard. The reference criteria were “current major depressive episode” or “current major depressive episode with major depressive disorder”. PHQ-9 was used with two thresholds: diagnostic algorithm and summary scores (PHQ-9 ≥ 10). The optimal cut-off points of PHQ-9 summary scores were analyzed using a receiver operational characteristics (ROC) curve.

**Results:**

For “current major depressive episode”, PHQ-9 showed high sensitivity and high negative predictive value at both thresholds, but its specificity and positive predictive value were low. For “current major depressive episode with major depressive disorder”, PHQ-9 also showed high sensitivity and high negative predictive value at both thresholds, but the positive predictive value decreased more than that for “current major depressive episode”. The ROC analysis showed the optimal cut-off score of 13/14 for “current major depressive episode”.

**Conclusions:**

PHQ-9 is useful for screening, but not for diagnosis of “current major depressive episode” in a psychiatric specialty clinic.

## Background

The Patient Health Questionnaire (PHQ) was developed in 1999 as a self-report version of the Primary Care Evaluation of Mental Disorders (PRIME-MD), which was designed for criteria-based diagnosis of several mental disorders that are commonly encountered in primary care [1]. The diagnostic validity of the depression module of the PHQ (PHQ-9) has been reported in a study involving 3000 patients in eight primary care clinics. Moreover, PHQ-9 for the diagnosis of major depressive disorder and any mood disorder showed moderate agreement with the diagnosis of the Structured Clinical Interview for *DSM-III-R* (SCID) by mental health professionals [1]. The validity for screening against the *DSM-IV* diagnosis of major depression, reliability, and feasibility of PHQ-9 is regarded as excellent [2]. PHQ-9 consists of the nine diagnostic criteria items of *DSM-IV* major depressive episode. Therefore, it has good logical validity for the *DSM-IV* diagnosis of major depressive episode.

Two recent meta-analyses have shown good pooled sensitivity (0.80 and 0.77, respectively) and specificity (0.92 and 0.94, respectively) for PHQ-9 against the *DSM-IV* diagnosis of major depressive disorder or major depressive episode in the settings of primary care clinics and clinics other than psychiatric clinics [3,4]. However, the utility and validity of PHQ-9 for the screening for major depressive episode or major depressive disorder has not been examined in a psychiatric specialty clinic. Although one might argue that such a brief self-report screening test is not necessary for specialist psychiatrists, various self-administered questionnaires are often used before a psychiatric interview, even in clinics specializing in psychiatric treatment. Above all, the screening for major depressive episode, which has a high prevalence, using a self-administered questionnaire such as PHQ-9 is useful to prevent underdiagnosis in patients with several other psychiatric disorders that might have comorbidity with major depressive episode. Therefore, the utility and limitation of PHQ-9 for patients with psychiatric disorders in psychiatric specialty clinics must be studied before its application to psychiatric practice.

For this study, a mood disorder specialist psychiatrist administered PHQ-9 to patients of psychiatric specialty clinics of a university hospital, and compared its diagnostic capability with that of *DSM-IV-TR* for diagnosis of major depressive episode and major depressive disorder. In earlier studies in primary care clinics and clinics other than psychiatric clinics, the *DSM-IV* diagnosis was done using SCID by trained interviewers or mental health professionals, but not by psychiatrists [1,5,6], which might be a disadvantage of earlier studies because psychiatric diagnosis, especially of hypomanic episodes, a history of which has a major impact on the accuracy of PHQ-9 screening for major depressive disorder, is extremely difficult, demanding many years of training. Additionally, we evaluated and discussed diagnostic factors contributing to false-positive diagnosis.

## Methods

### Subjects

From February 2008 to July 2009, 153 outpatients, who visited the Department of Psychiatry, Hokkaido University Hospital as new patients, were consecutively included in the study. Each had been diagnosed using the *Diagnostic and Statistical Manual of Mental Disorders, Fourth Edition, Text Revision (DSM-IV-TR)*[7] by a mood disorder specialist psychiatrist (T.I.) who was blinded to the PHQ-9 results and who had more than 20 years of clinical experience in this field of psychiatry. The Japanese version of PHQ-9 [8] was administered to patients during their waiting time as a routine clinical task. This study was performed in accordance with the Declaration of Helsinki and was approved by the institutional review board of Hokkaido University Hospital.

### PHQ-9

The PHQ-9 was self-completed by the patient in written form. Major depressive episodes were diagnosed in two ways using the PHQ-9: diagnostic algorithm and a summary score. The diagnostic algorithmic threshold for diagnosing major depressive episode was regarded as fulfilled if the answer to question #1a or question #1b and five or more of questions #1a–#1i was at least “more than half the days” (question #1i was counted if present at all) [1]. The threshold PHQ ≥ 10 signified that the summary score of questions #1a–#1i (range = 0–27) must be 10 or higher. This cutoff point was chosen because it has been reported and recommended the most consistently [3,4,6].

### Psychiatric evaluations

*DSM-IV-TR* diagnoses of various psychiatric disorders including mood disorders (major depressive disorder, minor depressive disorder, bipolar disorder, dysthymic disorder, and cyclothymic disorder) were made by a psychiatrist specializing in mood disorder (T.I.) using the *Quick Reference to Diagnostic Criteria from the DSM-IV-TR* on the same day when the patients answered PHQ-9. The average interview duration was 60 min. In each case, the presence of a current major depressive episode, which can appear in patients with major depressive disorder or bipolar disorder, was identified. In most patients with major depressive disorder, the 17-item Hamilton Depression Rating Scale (HDRS) and the Montgomery–Åsberg Depression Rating Score (MADRS, 10 items) were administered to evaluate the severity of depression in routine clinical work [9,10]. We also used the global assessment of functioning (GAF) scale, which reflects the overall level of psychological, social, and occupational functioning of individuals [7]. Some patients, however, did not receive the HDRS, MADRS, or GAF evaluation because of the limited time of the regular clinical interview.

### Data analysis

With respect to criterion validity, we investigated sensitivity, specificity, positive predictive value, negative predictive value, and the overall accuracy for the diagnostic algorithmic threshold and summary score threshold (PHQ ≥ 10). The *DSM-IV-TR* diagnoses of “current major depressive episode” and “current major depressive episode with major depressive disorder” were the criterion standard.

Receiver Operating Characteristics (ROC) analysis was used to ascertain an optimal cut-off point of PHQ-9 summary scores for screening for major depressive episode in the setting of a psychiatric specialty clinic. This curve shows “sensitivity” versus “1-specificity” for every possible cut-off point. The area under the curve (AUC) was calculated as an indicator of the discriminative property of the scale. A maximal “Youden Index” (sensitivity + specificity – 1) was calculated and suggested as an optimal cut-off point for PHQ-9 [11].

Pearson’s correlation coefficients between PHQ-9 scores and GAF or MADRS or HDRS scores of patients with major depressive disorder were calculated to assess their validity for severity measures of depressive symptoms.

All continuous data are presented as means with standard deviations or 95 % confidence intervals (CIs).

## Results

### Demographic characteristics and DSM-IV-TR diagnosis of subjects

Demographic characteristics and *DSM-IV-TR* diagnoses of 153 subjects are presented in Table 1. The most common diagnosis was major depressive disorder with subsequent bipolar disorder: 45 % of subjects were diagnosed with mood disorders. Two patients with major depressive disorder had comorbid psychiatric disorders: mild dementia and social anxiety disorder. Among them, only one patient with social anxiety disorder had a current major depressive episode that fulfilled its diagnostic criteria.

**Table 1:** **Characteristics and*DSM-IV-TR*diagnoses of 153 patients**

**Characteristic**	**Value**
Sex	
Female, *n* (%); male, *n* (%)	96 (63); 57 (37)
Age, mean ± SD (yr)	44.6 ± 18.3
Range	14–82
*DSM-IV-TR* diagnosis, n (%)	
Major Depressive Disorder	50 (32.7)
Minor Depressive Disorder	2 (1.3)
Dysthymic Disorder	1 (0.7)
Bipolar Disorder	16 (10.5)
Developmental Disorders	2 (1.3)
Dementia	10 (6.5)
Mental Disorders due to a General Medical Condition	7 (4.6)
Substance-Related Disorders	3 (2.0)
Schizophrenia	8 (5.2)
Schizoaffective Disorder	2 (1.3)
Panic Disorder	10 (6.5)
Other Anxiety Disorders	10 (6.5)
Somatoform Disorders	2 (1.3)
Eating Disorders	6 (3.9)
Insomnia	11 (7.2)
Adjustment Disorders	7 (4.6)
Premenstrual Dysphoric Disorder	1 (0.7)
None	5 (3.3)
Comorbidity with Major Depressive Disorder, currently met for a Major Depressive Episode	
Social Anxiety Disorder	1 (0.7)

### Validity of PHQ-9 for the screening for current major depressive episode

Table 2 (left half) presents operating characteristics of PHQ-9 for “current major depressive episode” for the PHQ-9 diagnostic algorithmic threshold and for the cut-off point (threshold) of summary score of PHQ-9 ≥10, which has been recommended most consistently as the cut-off point [3,4,6]. Both thresholds had satisfactory sensitivity and negative predictive value: the summary score threshold (PHQ ≥10) had slightly higher sensitivity (0.94) and a more negative predictive value (0.94) than the diagnostic algorithmic threshold did, although the former had slightly lower specificity and positive predictive value than the latter did.

**Table 2:** Sensitivity, specificity, positive and negative predictive values, and positive and negative likelihood ratios, and overall accuracy for the diagnostic algorithm and the summary score (≥10) of the PHQ-9

	**Diagnosis of current major depressive episode (MDE)**		**Diagnosis of MDE with major depressive disorder**	
	**Diagnostic algorithm of PHQ-9**	**Summary score (≥10) of the PHQ-9**	**Diagnostic algorithm of PHQ-9**	**Summary score (≥10) of the PHQ-9**
True positive, *n* (%)	39 (25)	47 (31)	32 (21)	38 (25)
False positive, *n* (%)	34 (22)	52 (34)	41 (27)	61 (40)
False negative, *n* (%)	11 (7)	3 (2)	9 (6)	3 (2)
True negative, *n* (%)	69 (45)	51 (33)	71 (46)	51 (33)
Sensitivity (95%CI)	0.78 (0.64–0.88)	0.94 (0.84–0.99)	0.78 (0.62–0.89)	0.93 (0.80–0.98)
Specificity (95%CI)	0.67 (0.57–0.76)	0.50 (0.40–0.60)	0.63 (0.54–0.72)	0.46 (0.36–0.55)
Positive predictive value (95%CI)	0.53 (0.41–0.65)	0.47 (0.37–0.58)	0.44 (0.32–0.56)	0.38 (0.29–0.49)
Negative predictive value (95%CI)	0.86 (0.77–0.93)	0.94 (0.85–0.99)	0.89 (0.80–0.95)	0.94 (0.85–0.99)
Positive likelihood ratio (95%CI)	2.36 (1.76–2.99)	1.86 (1.56–2.02)	2.13 (1.59–2.63)	1.70 (1.41–1.85)
Negative likelihood ratio (95%CI)	0.33 (0.19–0.53)	0.12 (0.04–0.33)	0.35 (0.19–0.59)	0.16 (0.05–0.43)
Overall accuracy (95%CI)	0.71 (0.64–0.76)	0.64 (0.58–0.67)	0.67 (0.61–0.72)	0.58 (0.53–0.61)

95%CI, 95% confidence intervals; MDE, major depressive episode.

Among 73 positive cases for the PHQ-9 diagnostic algorithmic threshold, 34 cases (46.6 %) were falsely positive for having a current major depressive episode, as inferred from low specificity and positive predictive value. The *DSM-IV-TR* diagnoses of false-positive cases were schizophrenia in 4 cases, panic disorder in 4 cases, adjustment disorder in 5 cases, eating disorders in 5 cases, dementia in 2 cases, and insomnia in 5 cases. The false-positive patients showed high PHQ-9 summary scores comparable to those of the true-positive patients (data not shown) and exhibited depressed mood, but their depressive symptoms were not continuous during more than 2 weeks, as defined in *DSM-IV-TR*. Only 2 patients were diagnosed with other depressive disorders (minor depressive disorder and dysthymia).

False-negative cases were far fewer than false-positive cases. This tendency was particularly pronounced for the threshold of the summary score of PHQ-9 ≥10

### Validity of PHQ-9 for the screening for current major depressive episode with major depressive disorder

Table 2 (right half) presents operating characteristics of PHQ-9 for “current major depressive episode with major depressive disorder” for the PHQ-9 diagnostic algorithmic threshold and for the cut-off point (threshold) of summary score of PHQ-9 ≥10. Both thresholds had satisfactory sensitivity and negative predictive value: the summary score threshold (PHQ ≥10) had slightly better sensitivity (0.93) and negative predictive value (0.94) than the diagnostic algorithmic threshold did, although the former had slightly lower specificity and positive predictive value than the latter did.

Compared with false-positive cases for the PHQ-9 diagnostic algorithmic threshold against “current major depressive episode”, the number of those against “current major depressive episode with major depressive disorder” increased by 7 patients. That is to say, the positive predictive value was decreased more than that for “current major depressive episode”. All of these additional false-positive cases were bipolar disorder patients: 1 bipolar I and 6 bipolar II patients. As expected, bipolar depressed patients were positive for PHQ-9 because depressive symptoms of major depressive disorder and bipolar disorder show only subtle differences [7,12].

### Receiver Operating Characteristics (ROC) analysis of the cut-off point (threshold) of summary score of PHQ-9 against “current major depressive episode”

**Figure 1:**
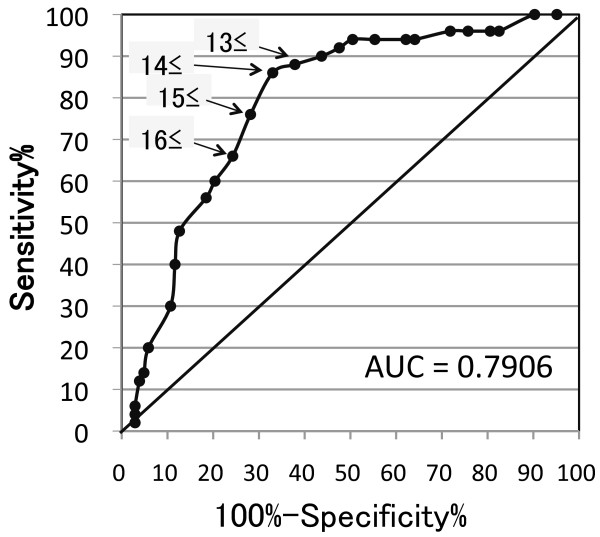
**Receiver operating characteristics (ROC) curve for the summary score threshold of the PHQ-9.** AUC, area under the curve.

Figure 1 displays the ROC curve of the cut-off point (threshold) of the summary score of PHQ-9 against “current major depressive episode”. The area under the curve (AUC) of 0.79 shows that the PHQ-9 summary scores can discriminate moderately between subjects with and without current major depressive episode [11]. Maximal discrimination between the presence and absence of “current major depressive episode” is achieved at the cut-off point which has the highest sum of sensitivity and specificity, as indicated by the “Youden index”. The optimal cut-off was 13/14 (sensitivity 0.86, specificity 0.67) in the setting of a psychiatric specialty clinic and higher than that (9/10) recommended in primary care facilities and other specialty clinics [3,4,6]. Screening purposes demand high sensitivity and negative predictive value. Such was the case for a cut-off score of 11/12 or lower (Table 3). For diagnostic purposes, high specificity and positive predictive value are necessary. However, this condition was not reached with any cut-off score because specificity was higher than 90 % with a cut-off score of 21/22 or higher, but positive predictive values of all cut-off scores were lower than 65 %.

**Table 3:** Sensitivity, specificity, positive and negative predictive values (PPV and NPV) at different cut-off scores for PHQ-9 summary scores against “current major depressive episode”

**Cut-off**	**Sensitivity**	**Specificity**	**PPV**	**NPV**
7/8	0.94	0.38	0.42	0.93
8/9	0.94	0.45	0.45	0.94
9/10	0.94	0.50	0.47	0.94
10/11	0.92	0.52	0.48	0.93
11/12	0.90	0.56	0.50	0.92
12/13	0.88	0.62	0.53	0.91
13/14 *	0.86	0.67	0.56	0.91
14/15	0.76	0.72	0.57	0.86
15/16	0.66	0.76	0.57	0.82
16/17	0.60	0.80	0.59	0.80
17/18	0.56	0.82	0.60	0.79
18/19	0.48	0.87	0.65	0.78
19/20	0.40	0.88	0.63	0.75
20/21	0.30	0.89	0.58	0.72
21/22	0.20	0.94	0.62	0.71
22/23	0.14	0.95	0.58	0.70

* Denotes the maximum sum of sensitivity and specificity.

### Correlation between PHQ-9 summary scores and HDRS, MADRS, or GAF

In patients with major depressive disorder, PHQ-9 summary scores were positively correlated with the severity measure of the depressive symptoms, HDRS and MADRS scores, but were negatively correlated with the overall levels of psychological, social, and occupational functioning, GAF scores [HDRS, *r* = 0 .55, 95%CI (0.30, 0.73), *P* = 0.0002, *N* = 42; MADRS, *r* = 0.68, 95%CI (0.45, 0.82), *P* < 0.0001, *N* = 36; GAF, *r* = −0.59, 95%CI (−0.78, -0.30), *P* = 0.0005, *N* = 31].

## Discussion

This study revealed that PHQ-9 has high sensitivity and a high negative predictive value in the setting of a clinic specializing in psychiatry as well as in primary care facilities and other specialty clinics [1,3,4,6] so that PHQ-9 is useful for screening purposes for the presence of a current major depressive episode. However, low specificity and a low positive predictive value in the setting of a clinic specializing in psychiatry do not support the use of PHQ-9 for diagnostic purposes in contrast to the setting of primary care facilities and other specialty clinics, for which high specificity and a high positive predictive value were reported [1,3,4,6,8]. These findings were shown similarly for both the diagnostic algorithmic threshold and the summary score threshold (PHQ ≥10), which were recommended for diagnostic and screening purposes by earlier studies [1,6].

Because of low specificity in both the diagnostic algorithmic threshold and the summary score threshold (PHQ ≥10), false-positive cases should be noted in the use of PHQ-9. For a current major depressive episode, schizophrenia, panic disorder, adjustment disorder, eating disorders, dementia, and insomnia were diagnoses of false-positive cases. Compared with patients visiting primary care and other specialty clinics, more patients with various psychiatric disorders showing depressed mood and other various symptoms visit clinics specializing in psychiatry, which engenders more false-positive results.

PHQ-9 is used primarily for screening for the presence of major depressive episode but not major depressive disorder because the *DSM-IV-TR* diagnosis of major depressive disorder demands several exclusion criteria such as the absence of manic or hypomanic episode, but PHQ-9 does not include such exclusion items [4]. A significant number of bipolar disorder patients are invariably misdiagnosed with major depressive disorder by PHQ-9 because a major depressive episode is part of bipolar disorder if one uses PHQ-9 for the screening for major depressive disorder. Originally, Kroenke et al. noted that before making a final diagnosis, the clinician is expected to rule out physical causes of depression, normal bereavement, and history of a manic episode [6]. Especially for a psychiatric specialty clinic, where bipolar disorder is much more prevalent, Kroenke’s notion must be considered. For this reason, we compared the operational characteristics of PHQ-9 against “current major depressive episode” and “current major depressive episode with major depressive disorder”. Our analysis of the validity of PHQ-9 for the screening for current major depressive episode with major depressive disorder (Table 2) revealed that the positive predictive value decreased by about 10 % compared with the screening for current major depressive episode. The underdiagnosis of bipolar disorder by PHQ-9 was a main reason for the increased false positives. Further diagnostic workup for past manic or hypomanic episodes or the combination of other screening tools for these episodes can resolve this major disadvantage of PHQ-9.

One might expect that the diagnostic algorithm threshold has better specificity than that of the summary score threshold because the diagnostic algorithm closely mimics the *DSM-IV-TR* diagnosis criteria of major depressive episodes. Nevertheless, against our expectations, the results obtained in this study and previous studies [3,4] showed no marked difference between two thresholds in operational characteristics. The summary score threshold (PHQ ≥10) has slightly higher sensitivity and negative predictive value, but slightly lower specificity and positive predictive value than the diagnostic algorithm with no marked difference in this study and a previous study of primary care [4]. The ROC analysis of the cut-off point (threshold) of summary score of PHQ-9 against “current major depressive episode” showed that the optimal cut-off was 13/14, which showed 0.86 of sensitivity and 0.67 of specificity comparable to those of the diagnostic algorithm, in the setting of a psychiatric specialty clinic. Table 3 shows that high specificity (>90 %) was reached with a cut-off score of 21/22 or higher, but a high positive predictive value was not reached with any cut-off score. The salient implication is that PHQ-9 used in a psychiatric specialty clinic might be suitable for screening purposes with the optimal cut-off of 13/14 of the summary scores for major depressive episode, but not for diagnostic purposes. The summary score threshold with different cut-off points for specific purposes might be preferred to the diagnostic algorithm.

Summary scores of PHQ-9 in patients with major depressive disorder were moderately correlated with severity measures of the depressive symptoms, HDRS and MADRS scores, positively, and with the overall levels of psychological, social, and occupational functioning, GAF scores, negatively. Consistent with our results, an earlier report of primary care described that PHQ-9 scores were correlated linearly with measures of quality of life, self-reported disability days, clinical visits, and self-reported difficulties related to symptoms [6]. In depressive disorder patients of primary care facilities, PHQ-9 scores were correlated moderately with the HDRS (17 items) scores [13]. In this study, the correlation of PHQ-9 scores was highest with the MADRS, which is related to the core concept of depression and which showed about twice the precision in estimating depression as the HDRS (17 items) showed for the average severity of depression [14]. Therefore, PHQ-9 scores might reflect the core symptoms of major depressive disorder, as inferred from items corresponding to the *DSM-IV* criteria items. To date, the correlation of PHQ-9 scores with the standard rating scales of depression, especially the MADRS, has not been reported in psychiatric patients. In addition to screening, PHQ-9 might be useful for measuring the severity of major depressive disorder.

All subjects in this study were psychiatric patients of a university hospital that provides primary and secondary services in Japan. These patients might have more complicated backgrounds than patients in other psychiatric clinics. Accordingly, these findings might not be generally applicable to other populations, which constitutes one limitation of this study.

## Conclusions

PHQ-9, a brief questionnaire, reportedly has excellent reliability and validity for screening of major depressive episode and for measuring the severity of depressive symptoms [2]. It has been used in primary care and medical specialty clinics. Results of this study suggest that PHQ-9 is useful also in clinics specializing in psychiatry. This report is the first of a study assessing the validity of the use of PHQ-9 in such clinics. However, caution is necessary for the use of PHQ-9 in psychiatry because PHQ-9 is useful only for screening purposes for “current major depressive episode” as a result of its low positive predictive value. The cut-off point 13/14 of PHQ-9 summary scores, which is higher than that (≥10) recommended in primary care, was optimal [6].

## Competing interests

The authors report no financial or other relationship that is relevant to the subject of this article. TK has received honoraria from GlaxoSmithKline, Astellas, and Eli Lilly, has received research/grant support from Astellas and GlaxoSmithKline, and is a member of the advisory boards of GlaxoSmithKline and Mitsubishi Tanabe Pharma. The other authors declare that they have no actual or potential conflict of interest.

## Authors’ contributions

TI designed the study and wrote the protocol. All other authors followed up and evaluated patients, and checked the protocol and discussion. All authors contributed to and approved the final manuscript.

## Pre-publication history

The pre-publication history for this paper can be accessed here:

http://www.biomedcentral.com/1471-244X/12/73/prepub
